# Clinical evaluation of fenestration decompression combined with secondary curettage for ameloblastoma of the jaw: retrospective radiographic analysis

**DOI:** 10.1186/s12903-022-02474-x

**Published:** 2022-10-14

**Authors:** Kailiu Wu, Hao Luo, Zhuang Yuan, Yanan Wang, Xing Qin, Jie He

**Affiliations:** 1 Department of Oral and Maxillofacial-Head and Neck Surgery, Shanghai Ninth People’s Hospital, Shanghai Jiao Tong University School of Medicine, 639 Zhizaoju Road, Shanghai, 200011 People’s Republic of China; 2 College of Stomatology, Shanghai Jiao Tong University, Shanghai, 200011 People’s Republic of China; 3 National Center for Stomatology, National Clinical Research Center for Oral Diseases, Shanghai, 200011 People’s Republic of China; 4 Shanghai Key Laboratory of Stomatology, Shanghai, 200011 People’s Republic of China; 5 Department of Stomatology, the Affiliated People’s Hospital of Jiangsu University, Zhenjiang, 212002 Jiangsu Province People’s Republic of China; 6 School of Stomatology, Xuzhou Medical University, Xuzhou, 221000 People’s Republic of China

**Keywords:** Ameloblastoma, Decompression, Curettage, Recurrence, Retrospective study

## Abstract

**Background:**

Ameloblastoma is a benign odontogenic epithelial tumor with local infiltration and a high recurrence rate that occurs most frequently in the jawbone. The aim of this study was to investigate the outcomes of fenestration decompression combined with secondary curettage (FDSC) in the surgical treatment of jaw ameloblastoma, and clarify the possibility of FDSC to become an appropriate therapeutic method for ameloblastoma with large lesion.

**Methods:**

A retrospective analysis was carried out in 145 patients diagnosed with multicystic ameloblastoma (MA) and 88 patients with unicystic ameloblastoma (UA). These patients were divided into two groups based on the therapeutic regimen: the FDSC group and the local curettage (LC) group. Panoramic radiography was taken 2 years after curettage to evaluate the change in lesion area in each case, and the therapeutic effects of different treatment methods were further assessed by the chi square test.

**Results:**

For MA, the effective rate of cystic cavity area reduction in the FDSC group (71.19%) was higher than that in the LC group (30.23%) (*P* < 0.001). For UA patients, the effective rate of lesion area reduction after FDSC was 93.02%, which was higher than that after LC (53.33%) (*P* < 0.001). Moreover, the recurrence rate of the FDSC group in the MA was 30.51%, which was significantly different from that of the LC group (*P* < 0.001). Regarding UA, the recurrence rates were 13.95% and 28.89%, after FDSC and LC, respectively, with no significant differences between the two groups (*P >* 0.05).

**Conclusions:**

FDSC exhibits a much better curative effect than LC in both MA and UA, whereas the recurrence rate of these two therapeutic strategies did not significantly differ in UA. The above data demonstrated that FDSC may serve as a routine, safe, effective and appropriate surgical treatment plan for MA or UA patients with large lesions.

## Background

Ameloblastoma, a benign odontogenic epithelial tumor with local infiltration and high recurrence rate, occurs most frequently in the jawbone, accounting for approximately 11% of all odontogenic tumors [1, 2]. It frequently occurs in young adults with no obvious difference between men and women according to the current literature [2–5]. The clinical manifestations of ameloblastoma include a painless expansion of the jawbone, a loosening or even loss of the teeth, a truncated absorption of the root, and facial deformity or asymmetry [6, 7]. Surgical treatment, the primary method for the treatment of ameloblastoma, can be divided into two categories: conservative and radical surgeries [8–10]. With the rise of functional surgery, an increasing number of surgeons tend to treat ameloblastomas by conservative means to retain the original jaw architecture and function, despite the higher recurrence rate after treatment [10–13]. Among these conservative surgical methods, fenestration decompression combined with secondary curettage (FDSC) and local curettage (LC) are the two most commonly used methods. FDSC has been reported to be more effective in treating unicystic ameloblastoma (UA) than LC [14, 15]. The benefits of FDSC for UA include avoidance of surgical damage to the jawbone, oral tissue preservation, retention of teeth and their vitality, and bone regeneration in the cystic cavity, etc. [16]. Hence, some clinicians have attempted to apply the FDSC method to multicystic ameloblastoma (MA), a subtype of ameloblastoma with aggressive biological behaviors and a higher recurrence rate [17]. However, these results seems to be not very optimistic because of the higher recurrence rate [12, 15]. Regarding the time consumption of FDSC and the limited number of MA cases, large sample data analysis is necessary, and more investigations should be implemented to assess the efficiency of FDSC in MA. LC and FDSC are two commonly used conservative surgical treatments for UA and MA at our hospital. Compared with LC, FDSC changes the internal environment of the tumor by decompression before curettage, which may be one of the reasons for the difference in treatment effect between these two methods. Moreover, we also want to understand the treatment effects of LC and FDSC in UA and MA, and the advantages of FDSC over LC.

In this study, we retrospectively analyzed the data of patients with MA and UA of the jaw treated with FDSC or LC to detect their shrinkage rate, compare their recurrence rates and detect the curative effect of FDSC in MA and UA, thereby clarifying the priority of FDSC in the treatment of UA and MA, and evaluating the possibility of FDSC to become a preferable treatment for ameloblastoma.

## Methods

### Ethical review and informed consent

This study was approved by the ethics committee of Ninth People’s Hospital of Shanghai JiaoTong University School of Medicine (Ref.SH9H-2021-T251-1), and the Helsinki Declaration guidelines were followed. Written informed consent was obtained from the patients who participated in the investigation.

### Study design and case selection

The protocol of this study was registered with ClinicalTrials.gov number NCT04987515. A retrospective chart review was carried out in MA and UA patients who received FDSC or LC treatment. Medical records of patients who were diagnosed with MA or UA by routine pathology were collected from January 2010 to December 2017 in Ninth People’s Hospital of Shanghai JiaoTong University Medical College. All eligible patients selected were divided into FDSC group and LC group. Demographic data such as age, gender, primary site and treatment effect were recorded. All patients were followed up for an average of 3–8 years. During the follow-up, panoramic radiography, maxillofacial CT or CBCT were taken.

### Surgical procedure

#### FDSC group

1. Reasonable design of the opening window: a window was designed in the oral cavity near the corresponding gingiva of the tumor, and part of the tissue was then excised for pathological examination. Subsequently, the rough bone surface was polished to be smooth. During the procedure, bone-cutting forceps or a power drilling system were used to open the bone compartment of all the cystic cavities to achieve unobstructed drainage. Following the completion of the window opening, the cystic cavity was thoroughly rinsed with a large amount of normal saline. The irrigation procedure was continued until a clear liquid was flushed out of the cystic cavity, and iodoform gauze was then stuffed into the cystic cavity.
2. Making a cyst plug: The iodoform gauze in the cystic cavity was removed within 3–7 days after surgery. Following the removal of the iodine gauze, an impression was taken to fabricate the cyst plug. The patient was asked to flush the cystic cavity more than 3 times a day, adjust the size of the cyst plug regularly, and take panoramic radiography to evaluate the changes in the area of the cystic cavity.
3. Patients returned every 3 months for follow up. If the cystic cavity area was gradually reduced within 1 year of follow-up, then these patients should be considered for secondary curettage surgery. If the tumor was enlarged, repeatedly infected, or caused osteomyelitis of the jaw, or if the open window was not normally occluded during the follow-up period, the follow-up observation was terminated immediately and further surgical treatment was carried out in time (these patients were not assigned to the FDSC group).
4. Secondary curettage: An incision was designed around the gingiva of the original opening window, followed by complete tumor clearance using a curette. Then, a dynamic drilling system was used to fully polish the bone walls around the cyst cavity to avoid missing tumor cells. The soft tissues at the gingival incision were partially released, and tight tension-free sutures were placed on the gingiva. Patients were still advised to undergo regular re-examination after surgery.

#### Local curettage group

The tumor was removed by local curettage only, and the specific operation method was the same as the that of secondary curettage.

### Outcome evaluation

All patients were re-examined every 3 months in the first year after surgery, and then every 6 months in the later period if the tumor had not recurred. Patients were followed up for more than 3 years, and panoramic radiography images were taken during the re-examination. The main evaluation indices included: (1) the change in the area of the cystic cavity involved in ameloblastoma; (2) tumor recurrence; and (3) tumor progression. Panoramic radiography taken 2 years after secondary curettage in the FDSC group or after curettage in the LC group was selected to measure the area of the cystic cavity. The area of the cystic cavity (mm^2^) = maximum horizontal distance (mm) × maximum vertical distance (mm). The percentage of cystic cavity area reduction = (preoperative cystic cavity area − postoperative cystic cavity area)/preoperative cystic cavity area × 100% [18]. When the area of the cystic cavity involved was reduced by at least 80%, the outcome was regarded as good. A reduction in area by at least 50% and less than 80% was considered a moderate effect. Treatment resulting in a reduction of the area of less than 50% was considered to be ineffective. Cystic cavity area reduction efficiency = (good effect case number + moderate effect case number)/total case number. All data were measured and calculated in triplicate by different investigators, and the average of the three measurements was taken to reduce the error.

### Statistical analysis

The data were statistically analyzed using the SPSS 21.0 software package, and discrete data are expressed as the number of cases (rate). Groups were compared with the chi-square test. The α level was set to 0.05 a priori, and *P* < 0.05 was considered statistically significant.

## Results

### General information of patients

A total of 233 eligible patients (145 patients with MA and 88 patients with UA) were screened out, and these patients were further divided into the FDSC group and LC group according to the treatment methods. The study population included 144 males and 89 females, with a male-to-female ratio of 1.62:1. Their ages ranged from 5 to 75 years old, with 59 patients aged 0 to 19 years old, 108 patients aged 20 to 39 years old, 51 patients aged 40 to 59 years old, and 15 patients older than 60 years old. The cohort included 13 cases of ameloblastoma in the maxilla and 220 cases in the mandible. The distribution of the patients in the FDSC group or LC group according to sex, age, anatomic location, pathologic subtype and therapeutic strategy is presented in Table 1. A statistical analysis showed a significant difference in the age distribution between the FDLC group and the LC group (*P* < 0.001).

**Table 1:** Sample demographics and baseline measures

Patients	FDSC group	LC group	*P*
Gender			0.992
Male	63	81	
Female	39	50	
Age			< 0.001
0 ~ 19	40	19	
20 ~ 39	48	60	
40 ~ 59	12	39	
≥ 60	2	13	
Position			0.122
Maxilla	3	10	
Mandible	99	121	
Classify			0.223
MA	59	86	
UA	43	45	

### Changes in the area of the cystic cavity involved in ameloblastoma

**Fig. 1:**
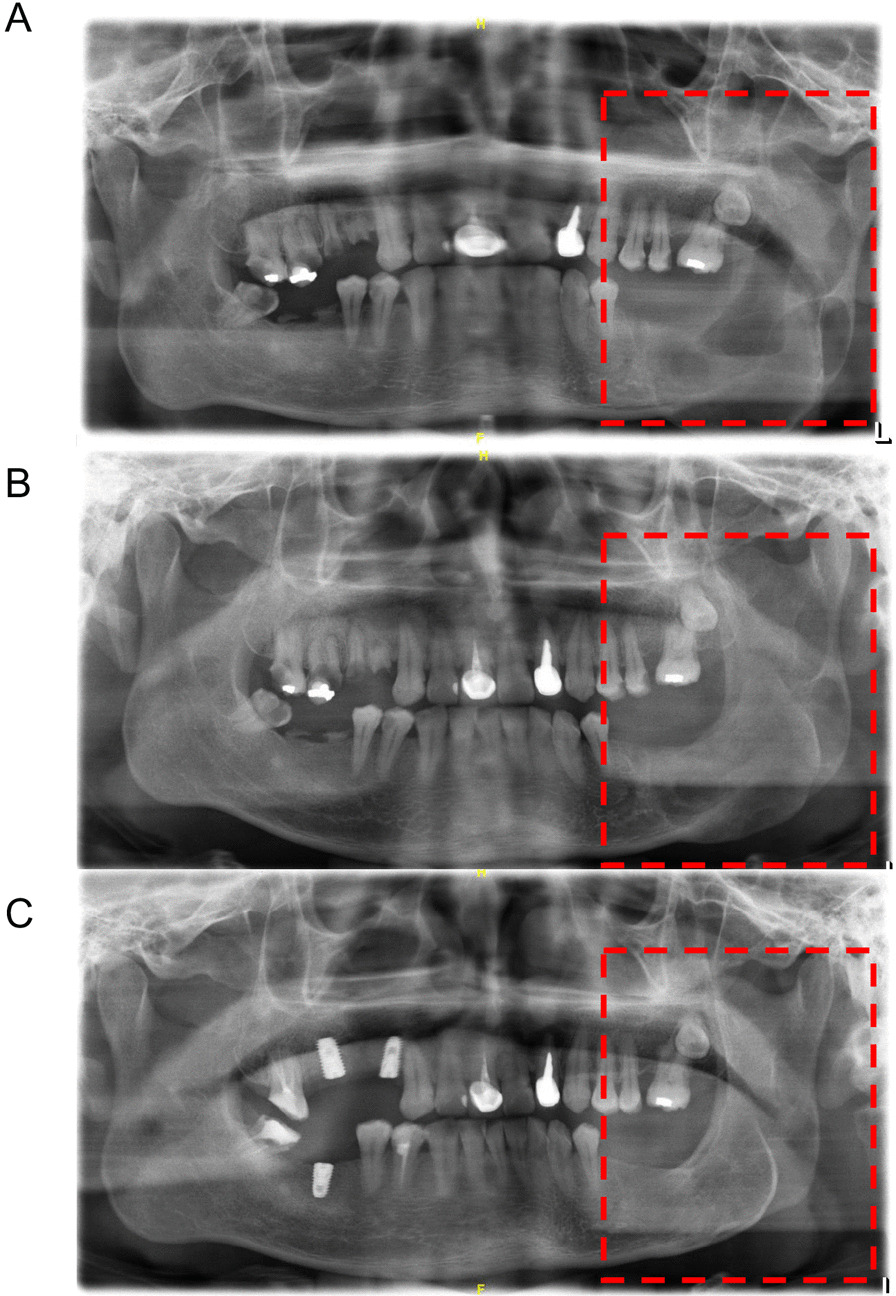
Comparison of pre-operative and post-operative results of FDSC strategy for MA patients. **a** Pre-operative panoramic radiography of patients; **b** The cystic cavity was reduced half a year after fenestration decompression; **c** The cystic cavity disappeared 2 years after secondary curettage

To evaluate the therapeutic effect of FDSC and LC in the treatment of MA or UA, the reduction rates of cystic cavity area were calculated based on radiological findings. Among the 145 cases of MA, 59 patients received FDSC treatment, and the cystic cavity area was reduced by more than 50% in 42 patients, corresponding to an effective rate of 71.19% (Table 2; Fig. 1). The LC group included 86 patients in total, and the cystic cavity area of 26 patients was reduced by more than 50% (the effective rate was 30.23%). Moreover, the reduction in cystic area significantly differed between the FDLC group and the LC group (Table 2, *P < *0.001).

**Table 2:** Comparison of the reduction rates of cystic cavity area after the surgical procedures of FDSC or LC in treatment of MA

Group(N)	Cystic cavity reduction effective(%)	χ^2^	*P*
FDSC group (59)	42 (71.19)	23.567	< 0.001
LC group (86)	26 (30.23)

**Fig. 2:**
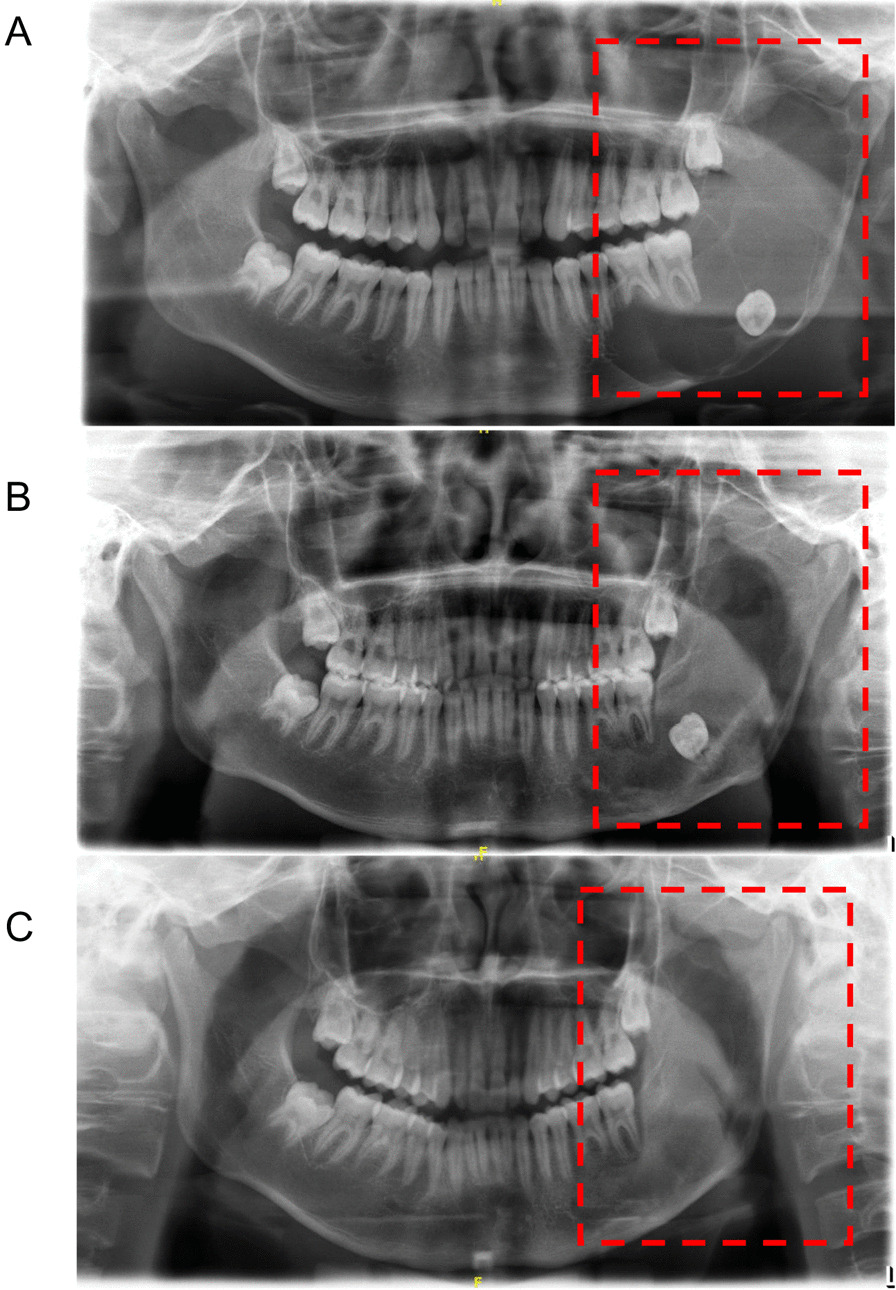
Comparison of pre-operative and post-operative results of FDSC in patients with UA. **a** Pre-operative panoramic radiography of patients; **b** The cystic cavity was reduced half a year after fenestration decompression; **c** The cystic cavity disappeared 2 years after secondary curettage

Among the 88 patients with UA, 43 cases of them were treated with the FDSC strategy, and the cystic cavity area of 40 patients was reduced by more than 50% (the effective rate was 93.02%; Fig. 2). In the LC group, the cystic cavity area of 24 patients was reduced by more than 50% (the effective rate was 53.33%). Furthermore, a statistical analysis showed that the reduction rate of the cystic cavity in the FDSC group was higher than that in the LC group (*P* < 0.001; Table 3).

**Table 3:** The effective rates of FDSC and LC for UA

Group(N)	Cystic cavity reduction effective(%)	χ^2^	*P*
FDSC group (43)	40 (93.02)	17.464	< 0.001
LC group (45)	24 (53.33)		

### Tumor recurrence

**Fig. 3:**
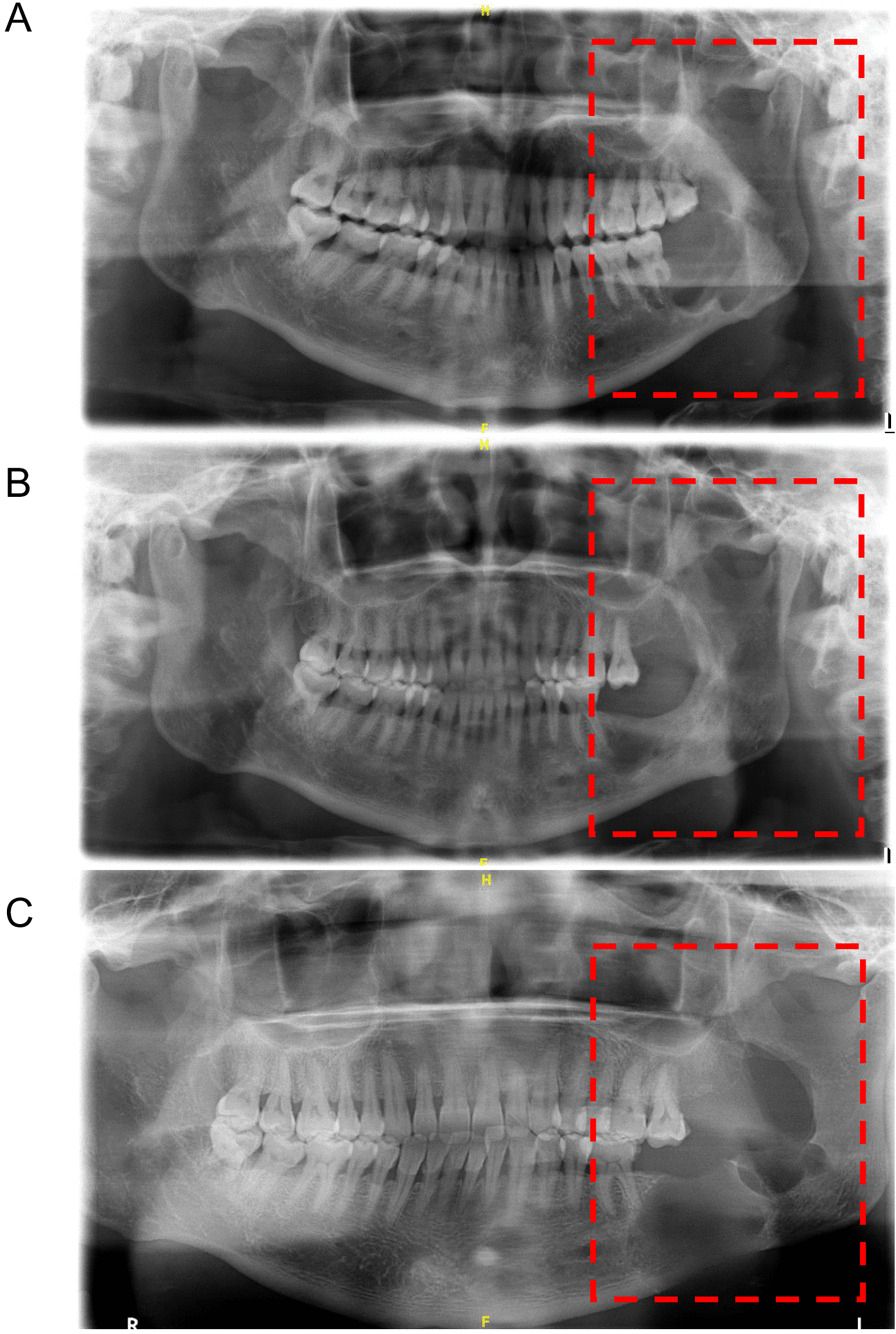
A case presentation of postoperative recurrence of MA. **a** Pre-operative panoramic radiography of patients; **b** The cystic cavity was reduced half a year after fenestration decompression; **c** Tumor recurrence 2 years after secondary curettage

Among 145 patients with MA, 59 patients received FDSC surgery. They were followed up after the surgery, and the tumor decreased in size. They were admitted to the hospital for secondary curettage, and postoperative follow-up was continued. In summary, 18 MA patients in the FDSC group experienced tumor recurrence, whereas 41 patients did not experience tumor recurrence (recurrence rate was 30.51%). Figure 3 shows a case of postoperative recurrence after the MA patient was treated with the FDSC method.

For the remaining 86 patients who received LC surgery, 55 patients experienced tumor recurrence (recurrence rate was 63.95%) after 3–8 years of follow-up. Approximately 37 patients with recurrence received LC treatment again, and 7 patients underwent segmental jaw resection after the failure of local curettage. The remaining 18 patients with tumor recurrence underwent direct radical surgery. Moreover, the cure rate of FDSC for MA was significantly higher than that of LC (*P* < 0.001, Table 4).

**Table 4:** The recurrence rate of ameloblastoma after two kinds of operation

Group(N)	Recurrence rate(%)	χ^2^	*P*
FDSC group (59)	18 (30.51)	15.658	< 0.001
LC group (86)	55 (63.95)		

**Fig. 4:**
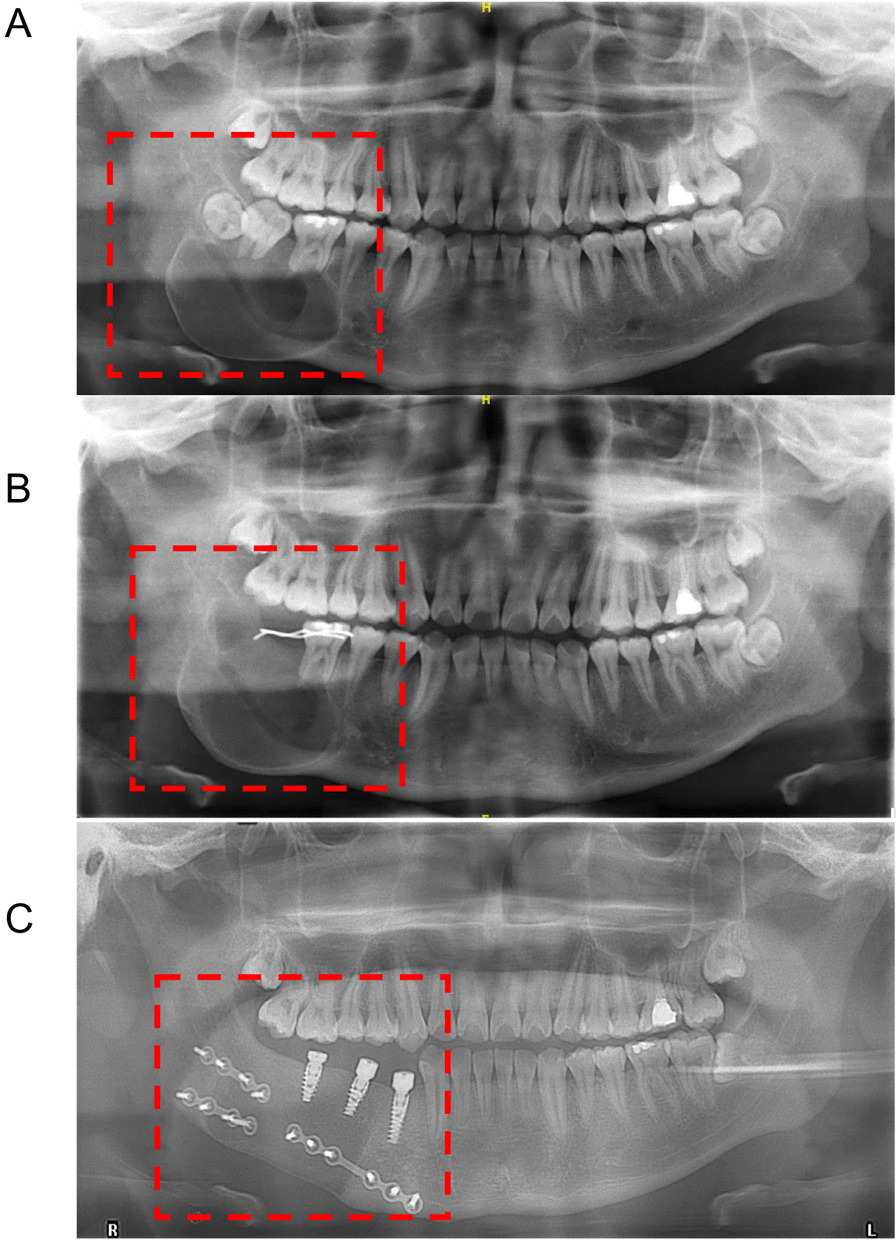
A case presentation of postoperative recurrence of UA. **a** Pre-operative panoramic radiography of patients; **b** Slight enlargement of the cystic cavity half a year after fenestration decompression; **c** A pantomography after the surgery of “Partial resection of right mandible + construction of vascularized free iliac myocutaneous flap”

Forty-three patients received FDSC treatment among the 88 patients with UA. They were followed up after fenestration decompression, and the area of the cystic cavity gradually decreased., The patients were finally admitted to the hospital for complete tumor curettage. Of these patients, 6 experienced recurrence after secondary curettage, and these patients were readmitted for radical surgery. Therefore, tumor recurrence occurred in 6 patients with UA after FDSC treatment (the recurrence rate was 13.95%). Figure 4 shows a panoramic view of a UA patient before the surgery, after recurrence and after radical reconstruction.

For the remaining 45 patients who received LC surgery, a total of 13 patients experienced tumor recurrence (the recurrence rate was 28.89%) during the follow-up. Among the patients with recurrence, 12 patients were admitted to the hospital for LC again, among whom 1 patient underwent segmental mandibular resection + vascularized free fibular musculocutaneous flap repair after failed LC. The remaining patient with recurrence received segmental resection of the jawbone plus adjacent tissue flap repair. A statistical analysis showed that the cure rate did not significantly differ between the two surgical procedures for UA (*P* = 0.089; Table 5).

**Table 5:** The recurrence rates of FDSC and LC for UA

Group(N)	Recurrence rate(%)	χ^2^	*P*
FDSC group (43)	6 (13.95)	2.897	0.089
LC group (45)	13 (28.89)		

### Malignant transformation

Through follow-up observation, 8 of 233 patients were found to have malignant transformation, and the malignant change rate was 3.43%. The cancerous component was the epithelial component of ameloblastoma, and the pathological tissue was finally diagnosed as squamous cell carcinoma. Malignant transformation was found in 2 patients who underwent FDSC and 6 patients who received LC treatment, but this difference was not significant. Moreover, all 8 patients had multiple recurrences after FDSC or LC surgery.

## Discussion

In the present study, we analyzed the effects of LC and FDSC in the treatment of UA and MA through a retrospective study. The results revealed that FDSC could shrink the cystic cavity more effectively and had a lower recurrence rate than LC. From the perspective of treatment effect and patient benefit, FDSC should be preferentially used in the clinical treatment of UA and MA.

The effectiveness of fenestration decompression in the treatment of odontogenic keratocysts and ameloblastomas has been widely recognized, especially for masses with a large range leading to severe deformation of the jaw [19, 20]. The purpose of fenestration decompression is to eliminate the conditions that are conducive to the continued expansion of the cyst [21]. Compared with partial jaw resection, this approach is easier and more conservative, with fewer perioperative complications, which is helpful to preserve the vitality of dental pulp, reduce the volume of lesions, and finally improve the quality of life of the patients [22–24]. After the tumor decreases in size, it can be cured by secondary curettage or excision. At the same time, we can also identify tumor residues in the local area by pathological detection. The above treatment process is the FDSC method mentioned in this study. FDSC is a commonly used conservative surgery in the treatment of ameloblastoma. Radical surgery is often more traumatic and invasive, and the tumor and part of the mandible can be completely removed beyond the safety margins through an expanded resection or segmental resection of the jaw above 1.0 cm ~ 1.5 cm in the lesion area. Compared with conservative surgery, radical surgery has a lower rate of tumor recurrence [25, 26]. However, long-term dentition loss and jaw defects always result in great inconvenience to these patients. Hence, an increasing number of researchers have favored conservative treatment methods, such as FDSC, to enable patients to improve the quality of life of patients. FDSC can maximize the tumor cure rate while eliminating obvious facial scarring and avoiding damage to the important structures of the jaw through the selection of an intraoral incision [17, 27, 28]. Although the maximum reported failure rate of FDSC for ameloblastoma of the jaw is 32.6% with FDSC, this procedure remains associated with a high probability of retaining the original jaw architecture and a high prognostic quality of life [29]. In the present study, the treatment efficiency of FDSC was 71.19% (42/59) for MA patients and 93.02% (40/43) for UA patients, corresponding to an overall effective rate of 80.39% (82/102). Moreover, FDSC was more effective for the treatment of UA patients than MA patients. The above data were in accordance with the conclusions of previous studies [11, 17, 29, 30]. As shown in Figs. 1 and 2, the lesion area was replaced by new bone, and the morphology of the mandible was well improved after FDSC treatment, which constitute advantages of FDSC over radical surgery.

Nevertheless, the FDSC method is also associated with limitations. Due to the limited exposure of the operative area, completely eradicating the tumor cells hidden in the ascomytes is not easy [31]. Moreover, new dead cavities might be formed in the cystic cavity during tissue remodeling, especially in MA. In addition to the above two factors, a large amount of residual tumor tissue after fenestration may also cause FDSC treatment to fail, and the recurrence of ameloblastoma was also closely associated with its pathological results. Approximately 50% of recurrent ameloblastomas have been reported to be of the follicular type, and this incidence is followed by those of the plexiform type, desmoplastic type and mixed type (consisting of follicular and plexiform type) [32, 33]. Hence, many researchers have attempted to identify measures to reduce the recurrence of ameloblastoma after FDSC such as carbolic acid washing, chemotherapeutic drug washing and cryotherapy [31, 34]. Other supplementary treatment measures include radiotherapy, chemotherapy and targeted directed therapies (targeting mutated BRAF, MEK or FGFR2) [31]. Considering the advantage of FDSC, methods to improve the FDSC method are very meaningful for ameloblastoma treatment. This study summarized cases in which FDSC failed, which highlighted the following: (1) The design of the window should be reasonable (appropriate size and location, removing the affected teeth if necessary) to ensure that the contents of the cyst fluid can be fully drained. (2) The effect of fenestration decompression was not ideal when the tumor had destroyed the bone cortex and the root absorption was apparent [16]; For MA, patients with a clear bony septum on imaging always had a good prognosis, and the premise was that the bony septum should be completely opened during surgery. (3) Protection area periosteal bone integrity should also be addressed. The integrity of the jaw bone periosteum, a physiological barrier, its integrity is very important for the reconstruction of jaw shape after fenestration decompression. (4) Patient compliance is very important. After fenestration, the capsule cavity needs to be rinsed every day, rechecked and photographed regularly so that the physician can timely evaluate the changes in the lesions and administer corresponding treatment measures in a timely manner. In addition, our research group slightly modified the FDSC procedure. For patients suitable for FDSC treatment, we not only performed fenestration but also simultaneous curettage, but the follow-up treatment remained consistent with that of FDSC. The purpose of this modification was to simultaneously reduce the tumor recurrence rate by decompression and ensure tumor reduction. At present, the study has yielded relatively optimistic preliminary results (data not shown), but the recurrence rate needs to be evaluated through long-term follow-up.

The recurrence rate of ameloblastoma treated with conservative methods also significantly differs between studies [26, 35]. Lau et al. reported that the recurrence rate after fenestration decompression was 18% for UA [36], Nakamura et al. summarized that 8 of 11 (72.7%) patients with MA who received FDSC had recurrences [11], whereas Yang et al. reported an overall recurrence rate of UA and MA of 4.5% (2/44) after marsupialization [17]. For conservative treatment, the recurrent rate reported by Goh et al. was 52% [32], while another study indicated a low recurrence rate of 11% for ameloblastoma patients treated with conservative treatment [34]. In the present study, the overall recurrence rate was 39.48% (92/233; including 19 patients with UA and 73 patients with MA); compared with UA patients (recurrence rate: 21.59%, 19/88), MA patients (recurrence rate: 50.34%, 73/145) were more likely to relapse after FDSC or LC treatment; moreover, the recurrence rate of patients treated with the FDSC method (recurrence rate: 23.53%, 24/102) was significantly lower than that of patients treated with the LC method (recurrence rate: 51.9%, 68/131). The trend of these data is consistent with that of previously reported data, but the recurrence rate was higher [11, 23, 36]. Several factors may explain this discrepancy. First, the observation period after fenestration or curettage was long, and many patients with better therapeutic effects did not come for follow-up visits or received secondary treatment, which reduced the number of patients with good treatment effects. Second, age was considered as an important risk factor for the recurrence of ameloblastoma. The reported results revealed that patients with ameloblastoma aged 21 to 40 years were more likely to develop recurrence [32, 37], and patients in this age group accounted for approximately 50% of the population in our study. Third, the follow-up time was relatively long. Most recurrence cases of ameloblastoma reportedly occur > 4 years after initial treatment [35]. In the present study, all patients were followed up for 3–8 years, and the mean follow-up time was much longer than 4 years. Therefore, the high recurrence rate after FDSC or LC can be considered reasonable.

In this study, 8 patients eventually developed cancer, all of whom were MA patients. Few reports currently describe the malignant transformation of ameloblastoma, and the postoperative recurrence rate is closely related to the treatment method. Yoon et al. [38] reported a 5-year overall survival rate of 72.9% for malignant ameloblastoma, a recurrence rate of 28.3% for patients who received radical resection, a recurrence rate of over 90% for patients who received curette or enucleation, and a survival rate of 21.4% for patients with metastasis. The recurrence rate of patients treated conservatively with chemotherapy and radiotherapy was 92.3%. In our study, all patients with cancer underwent radical surgery to remove the lesions after admission and were followed up for life. To date, 1 patient developed pulmonary metastasis, 2 patients relapsed with ipsilateral cervical lymph node metastasis, and the remaining 5 patients had no recurrence or metastasis. Patients with pulmonary metastasis were sent to thoracic surgery for radical resection of metastatic foci, and patients with recurrent metastasis were readmitted for radical resection + neck lymph node dissection. One patient with cervical lymph node metastasis received 30 courses of radiotherapy after radical neck lymph node dissection, and no recurrence has been observed at present. The cause of ameloblastoma malignant transformation is currently unclear and may be related to repeated inflammatory stimulation, stimulation by physical and chemical factors, or gene mutation etc. after fenestration or curettage.

Recently, interest in the development of clinical-pathological prognostic models for oral squamous cell carcinoma has increased, and such models could guide physicians to formulate correct treatment [39]. SMO gene mutation or CD44 dysregulation can reportedly contribute to a higher recurrence of ameloblastoma [40, 41]. However, few studies have reported risk genes that cause the malignant transformation of ameloblastoma. In future studies, technologies such as exon sequencing, RNA sequencing and protein mass spectrometry should be implemented to clarify the genes associated with the recurrence and malignant transformation of ameloblastoma. Physicians could then establish specific clinical-pathological prognostic models guiding them toward the correct treatment choice.

However, this study also has some limitations. One limitation of this study was the difference in the age composition between the FDSC group and LC group. This difference may be due to the a loss of follow-up. Since this study was a retrospective analysis, the treatment process may have also differed between patients with UA and patients with MA, which may eventually lead to a certain deviations in the data. Another limitation was that in this study, the evaluation of lesion ranges was based on panoramic films, which were not sufficiently accurate compared with CT. Hence, a reasonable study design (such as a case–control study) and accurate evaluation measures should be taken up to further assess the effect of the treatment for patients with ameloblastoma in future studies. Therefore, significant efforts should be made to improve the conservative measures for the treatment of ameloblastoma and optimize outcomes for patients.

## Conclusions

This study retrospectively analyzed the therapeutic effects of FDSC and LC in the treatment of MA and UA and summarized the recurrence rate and malignant transformation after treatment. The results showed that FDSC yielded a better therapeutic effect than LC in both MA and UA, and patients with MA were more likely to relapse after surgery. Moreover, the postoperative recurrence rate of patients treated with FDSC was significantly lower than that of patients treated with LC. These data revealed that FDSC might serve as a routine, safe and effective surgical treatment plan for MA or UA patients with large lesions. More importantly, with the aim to optimize therapeutic outcomes for patients, surgical regimen needs to be continuously improved in the future: optimization of surgical methods; development of comprehensive treatment strategies to increase the efficacy of surgical treatment through the combination with non-surgical treatment; establishment of individualized treatment plan based on molecular classification and the prediction of prognosis and recurrence of ameloblastoma.

## Data Availability

Data and materials are available and accessible from the corresponding author on reasonable request.
